# Progressive Ataxia, Memory Impairments, and Seizure Episodes in *Spna2 R1098Q* Mouse Variant Affecting Alpha II Spectrin’s Scaffold Stability

**DOI:** 10.3390/brainsci13020261

**Published:** 2023-02-03

**Authors:** Michał Zalas, Joanna Skrzymowska, Apolonia Miążek, Arkadiusz Miazek

**Affiliations:** 1 Department of Tumor Immunology, Hirszfeld Institute of Immunology and Experimental Therapy, Polish Academy of Sciences, Weigla 12, 53-114 Wroclaw, Poland; 2 Department of Pathomorphology, Faculty of Medicine, Jagiellonian University Medical College, Grzegorzecka 16, 31-351 Krakow, Poland; 3 Department of Biochemistry and Molecular Biology, Wroclaw University of Environmental and Life Sciences, Norwida 31, 50-375 Wroclaw, Poland

**Keywords:** *SPTAN1*, spectrinopathy, ataxia, seizure, alpha II spectrin, motor neuropathy

## Abstract

*SPTAN1* spectrinopathies refer to a group of rare, inherited diseases associated with damage to non-erythrocytic α-II spectrin (α-II). They are linked to a range of mild to severe neuropathologies of the central and peripheral nervous systems, such as early infantile epileptic encephalopathy type 5, cerebellar ataxia, inherited peripheral neuropathy, and spastic paraplegia. Modeling human *SPTAN1* encephalopathies in laboratory animals has been challenging partially because no haploinsufficiency-related phenotypes unfold in heterozygous *Spna2* deficient mice nor stable transgenic lines of mice mimicking missense human *SPTAN1* mutations have been created to date. Here, we assess the motor and memory performance of a dominant-negative murine *Spna2* (*SPTAN1*) variant carrying a spontaneous point mutation replacing an arginine 1098 in the repeat 10th of α-II with the glutamine (R1098Q). By comparing groups of heterozygous R1098Q mice at different ages, we find evidence for progressive ataxia, and age-related deterioration of motor performance and muscle strength. We also document stress-induced, long-lasting seizure episodes of R1098Q mice and their poor performance in novel object recognition memory tests. Overall, we propose that the complexity of neuropathology-related phenotypes presented by the R1098Q mice recapitulates a number of symptoms observed in human patients carrying *SPTAN1* mutations affecting α-II scaffold stability. This makes the R1098Q mice a valuable animal model for preclinical research.

## 1. Introduction

At least 40 known *SPTAN1* mutations with confirmed Mendelian inheritance patterns are described to date [1]. These mutations are linked to a spectrum of mild to severe phenotypes involving either central or peripheral neuropathology or both [2]. In their most severe form, epileptic encephalopathies (West syndrome) with developmental delay, accompanied by cerebellar atrophy, hypomyelination, and microcephaly, often lead to early infantile death [2]. Other phenotypes related to *SPTAN1* mutations include cerebellar ataxias, hereditary-motor and sensory-motor neuropathies, and spastic paraplegia [3,4]. Several molecular mechanisms were proposed to account for the large phenotypic spectrum of *SPTAN1* mutations. The nonsense-mediated decay of mRNA encoding mutated *SPTAN1* allele causes a decrease in the total expression level of α-II, believed to contribute to disease etiology [3]. Another mechanism, recognized in numerous dominant-negative *SPTAN1* mutations prevents the proper heterodimerization of α-II with beta spectrins and promotes the accumulation of cytotoxic α-II intracellular aggregates [5]. Another group of recently identified de novo missense *SPTAN1* mutations involve positively charged amino acids located at positions, interconnecting the three-helix bundle of spectrin repeats [1]. This set of mutations shares an otherwise unrecognized feature of affecting spectrins’ scaffold bending and flexibility.

Strong evolutionary conservation of spectrins’ primary amino-acid sequences makes mice relevant animal models of inherited human spectrinopathies [6,7]. Indeed, almost all studied loss-of-function variants of murine homologs of *SPTBN1*, *SPTBN2*, *SPTBN3,* and *SPTAN1* present phenotypes similar to the clinical manifestations in affected patients [8]. For instance, although homozygous *SPTBN1* mutant mice die in the midterm of gestation, the heterozygous neuron-restricted *SPTBN1* mutation in mice presents a number of characteristics observed in patients carrying heterozygous, dominant-negative *SPTBN1* variants, including impaired motor abilities, social and learning deficits (i.e., hyperactivity attention-deficit/hyperactivity disorder and autism spectrum disorder), facial dysmorphisms, arrested growth, and smaller weight [9]. The homozygous *SPTBN2* deficiency in mice does not cause premature embryonic death of animals, but it appears in a form of progressive gait abnormalities, tremors, Purkinje cell loss, and cerebellar atrophy (molecular layer thinning), recapitulating features of spinocerebellar ataxia type 5 in humans [10]. No ataxic phenotype, however, is observed in heterozygous mutant mice, arguing against haploinsufficiency as a disease mechanism. The “quivering” mice carrying recessively inherited loss-of-function mutations of murine *SPTBN3* homolog present auditory and motor neuropathies, similar to symptoms observed in *SPTBN3* homozygous or compound heterozygous patients suffering from congenital muscular hypotonia, neuropathy, and deafness [11,12]. General or organ-restricted gene ablation of *SPTAN1* ortholog in rodents uncovered its essential roles in the development and function of neural and cardiac systems and cochlear hair cell function [6,7,8,13].

The homozygous loss-of-function mutation of murine *Spna2* (*SPTAN1*) is lethal during embryonic development due to retarded intrauterine growth and craniofacial, neural tube, and cardiac anomalies [14]. In contrast to heterozygous *SPTBN1* mutant mice, the *SPTAN1* heterozygotes present no obvious phenotype, precluding their use as models for human pathogenic *SPTAN1* variants, whose phenotypic effects appear after birth. In this context, the identification of a spontaneous dominant-negative missense *SPTAN1* R1098Q variant in C57Bl/6J mice offers an experimental model of *SPTAN1* spectrinopathy unfolding during postnatal life [15].

Here, we aimed at further extending our initial observations on motor incoordination of R1098Q mice, and additionally assess their muscle strength, memory performance, and seizure episodes at different ages. Our analyses uncovered a number of phenotypic features of R1098Q mice that can be directly compared to clinical presentations in patients carrying pathogenic *SPTAN1* variants. Thus, R1098Q mice constitute a unique pre-clinical model for studying phenotypic and molecular aspects of dominant-types missense α-II mutations affecting spectrin’s repeat stability.

## 2. Materials and Methods

### 2.1. Mice

Animal experimentation whose severity qualified it as a procedure according to European Union directive on the protection of animals used for scientific purposes (2010/63/EU) was approved by the Local Ethical Committee in Wroclaw (Poland) under permission numbers 78/2018 and 70/2021. The Spna2R1098Q mice (R1098Q) were backcrossed for at least ten generations to C57Bl6/J (WT). The mice were divided into three age groups: young (8 wks old), adult (24 weeks old), and old (54 weeks old). All efforts were made to minimize the number of animals used for testing, and their distress, and suffering. Mice were housed in individually ventilated cages (Techniplast, Buguggiate (VA), Italy) (2–5 mice/cage) in air-conditioned rooms (temperature 22 ± 2 °C), with a controlled humidity level (55 ± 10%) and automatic regulation of the day/night cycle (12/12 h). Constant access to filtered and sterilized water and a standard sterilized rodent chow (Ssniff Spezialdiäten GmbH, Soest, Germany) was provided. Elements of environmental enrichment (wooden tunnels for abrasion of incisors, plastic domes, and materials to build lair and cardboard tubes to play) were provided in every cage. In 5–7 days before the planned experiments, the mice were tamed by handling. Handling activities were aimed at getting the animals used to picking up and taking into hands, and in the same sessions, the animals were getting used to the devices on which the experiments were conducted, as well as with the room where the tests were performed.

### 2.2. RotaRod

An in-house rotarod apparatus (designed and manufactured by Eng Robert Budziński) fitted with an automatic timer and falling sensor was used. Three constant rotational speeds were chosen for this study (4, 8, and 14 rpm). The cutoff time for a single trial was set to 120 s. Mice were given two practice trials, 48 and 24 h before the first session to habituate them to the test conditions. Then, each mouse had three trials per session, four sessions in total, with one session per day on four consecutive days. At least 3 min intervals between the trials were applied. The results of trials were averaged for analysis. If a mouse stopped walking (rotated passively) before reaching 120 s, it was counted as if it fell off the rod.

### 2.3. Parallel Rod Floor Test

The parallel rod floor test assesses unforced sensorimotor and balance deficits. The apparatus used for this test was constructed in-house (by Eng. Robert Budziński), according to published guidelines [16]. The 150 mm × 150 mm arena surrounded by 200 mm high Plexiglass walls was made with parallel metal rods (1,2 mm diameter) with inter rod distance of 0.75 cm. The apparatus was fitted with an automatic misstep counter, an infrared movement detector, and electronic timer. Mice were individually placed at the center of the grid and allowed to freely walk a total distance of 10 m. A number of missteps and time needed to travel this distance was recorded. The test was performed on 4 consecutive days, one trial per day.

### 2.4. Footprint Test

This test allows for a detailed analysis of motor coordination and gait during normal walking. Mice had their front and hind paws painted with non-toxic dyes (Winsor & Newton, London, UK) in green and black, respectively. The mice were then allowed to walk along a 300 × 100 mm runway into an enclosed box leaving their footprints on a sheet of Whatman filter paper (Whatman Ltd., Midestone, UK). The distances between the specific traces were measured (average values of at least 5 steps per mouse) to determine the following parameters: the stride length (the distance between successive contacts of the same paw), the step length (the distance between successive contacts of contralateral front or hind paws along the axis of the direction of motion), the base of support (BOS—a distance between successive contralateral front or hind paws perpendicular to the axis of direction of motion), and the interlimb distance (a distance between the ipsilateral front and hind paws).

### 2.5. Wire Hang Test

This test is best suited for measuring muscle coordination and endurance in rodents. The device used for this test consisted of a 2-mm thick, non-stretchable and non-vibrating, 350 mm long multistranded metallic wire (Inoxa Sp. z o.o., Jaworzno, Poland) stretched between two vertical stands of 300 mm high. On the bottom of the device, there was a layer of shock-absorbing bedding material to prevent injury of an animal when it fell. The mouse was hung at the center of the wire, and the time until it fell or until the test cutoff time (180 s) passed was measured.

### 2.6. Novel Object Recognition Test (NOR)

The NOR test was used for evaluating cognitive functions, in particular, recognition memory in mice. Each mouse was habituated to an empty arena (500 × 300 × 500 mm) one day before the test. At the day of the test (training session) each mouse was exposed to two identical objects placed at the center of arena, 150 mm apart, for 10 min. On the next day (test day) the mice were allowed to explore one unchanged and one new object for 10 min. The sessions were recorded using a camera (Xiaomi, Beijing, China) mounted above the arena. After each session, the arena and the objects were cleaned with distilled water and 70% ethanol to remove any residual scent. Exploration was defined as time spent pointing the head toward an object at a distance of 15 mm from the object. Climbing and sitting on the objects were not considered exploratory behaviors.

### 2.7. Behavioral Assessment of Seizure

The duration of handling-induced seizures was recorded with a digital timer after briefly turning the animal’s head down. The severity of rotarod-induced seizure episodes in R1098Q mice was assessed using an adapted Racine behavioral scale [17]. Seizure episodes were recorded with a digital camera (Xiaomi, Beijing, China) and analyzed for the presence of characteristic behavioral features. The prevalence and duration of observed behavioral features were shown in Supplementary Videos S1–S10 and summarized in Supplementary Table S1.

### 2.8. Statistical Analysis and Data Presentation

Data are presented as means ±SD. The Shapiro–Wilk test was performed to assess data normality. On this basis, either Student’s *t*-test, for pairwise comparisons, or one-way ANOVA for multiple comparisons of normally distributed values was used followed by Bonferroni post hoc test. For non-normally distributed values either the Mann–Whitney or the Kruskal–Wallis ANOVA test followed by Dunn’s post hoc test for multiple non-parametric comparisons was used. GraphPad Prism software v7 (Dotmatics, Boston, MA, USA) was used for the graphical representation of data.

## 3. Results

We have previously reported on the identification of alpha II (αII) spectrin mouse variant displaying progressive ataxia [15]. To further extend our initial observations we performed a battery of motor coordination tests on three groups of R1098Q mice (8, 24, and 54 weeks old) and, in parallel on sex and age-matched WT control littermates.

### 3.1. Rotarod

**Figure 1:**
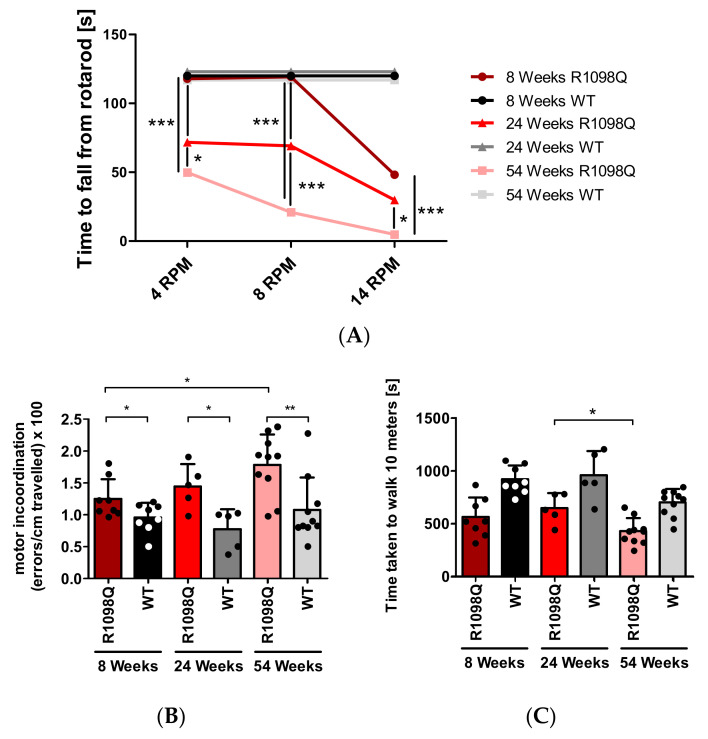
Progressive motor impairment in R1098Q mice: (**A**) Constant speed rotarod. The graph shows the mean time taken to fall off the rotarod for three age groups of R1098Q mice and control WT littermates. All the WT mice achieved a maximum latency of 120 s. The graphs of WT performance were separated for better readability of the results, otherwise, they would overlap. The numbers of animals per group were as follows: the 8-week-old group (WT, n = 5; R1098Q, n = 7), the 24-week-old group (WT, n = 7; R1098Q n = 7), the 54-week old group (WT, n = 6; R1098Q n = 7). (**B**) parallel rod floor test: (**C**) open field test. The bars represent the mean of the group, and the dots represent the mean result of four trials per mouse. The tests shown in panels B and *C* were performed on the following numbers of mice per group: the 8-week-old group (WT, n = 8; R1098Q, n = 8), the 24-week-old group (WT, n = 5; R1098Q n = 5), and the 54-week-old group (WT, n = 10; R1098Q, n = 10). Error bars represent standard deviations (SD). The statistics were calculated using the Kruskal–Wallis ANOVA (**A**), *t*-test, and Mann–Whitney test (**B**,**C**). Statistically significant differences between R1098Q mice at different ages indicating the progression of the phenotype are marked with asterisks. (* *p* < 0.05, ** *p* < 0.01, and *** *p* < 0.001).

When tested on constant speed rotarod, we observed a progressive decline in the performance of all tested age groups of R1098Q mice with increasing rotarod speeds (Figure 1A, and Supplementary Figure S1). Kruskal–Wallis ANOVA revealed that there was a significant interaction between the age of R1098Q mice, *H* (2, N = 60) = 26.824, *p* = 0.0001 and rotarod speed *H* (2, N = 60) = 16.384, *p* = 0.0003 on the time to fall off rotarod. When comparing R1098Q to WT controls, at lower rotarod speeds (4 and 8 rpm) the group of young (8 weeks old) R1098Q mice performed nearly as well as WT littermate controls, whereas at 14 rpm, the time to fall off rotarod was significantly shorter (Mann–Whitney *U* =0.00, n_1_ = 7, n_2_ = 5, *p* = 0.0042) (Supplementary Figure S1). Additionally, in the group of adult mice (24 weeks old) at 8 and 14 rpm, there was a significant difference in the time to fall off rotarod between R1098Q and WT genotypes (at 8 rpm: Mann–Whitney *U* = 3.5, n_1_ = 6, n_2_ = 7, *p* = 0.0081, at 14 rpm: *U* = 0.00, n_1_ = 6, n_2_ = 7, *p* = 0.0011). In the group of 54 weeks old mice at all rotarod speeds, we detected significant differences between the R1098Q and WT mice (at 4, 8, and 14 rpm: Mann–Whitney *U* = 0.00, n_1_ = 7, n_2_ = 6, *p* = 0.0011).

### 3.2. The Parallel Rod Floor Test

To assess the sensorimotor coordination of R1098Q mice in an unforced way, we performed a parallel rod floor test. Pairwise comparison of the numbers of foot slips per centimeter of the traveled distance between age-matched R1098Q and WT mice revealed that in every age group, the R1098Q mice had significantly more foot slips than WT mice (Figure 1B). However, consistently with rotarod results, in the case of young, 8-wk-old mice, the difference in the mean numbers of foot slips between the R1098Q (0.01250 ± 0.003070 errors/cm) and WT (00956 ± 0.00233 errors/cm) was barely significant (*p* = 0.0491) (Figure 1B). A progressive decline of performance measured as an increase in foot slips between younger and older mice revealed a significant difference between the group of 8 wks old and 54 wks old R1098Q mice (*p* = 0.0153), but no such significant difference between the group of 8 and 24-wk-old mice (Mann–Whitney *U* = 13, n_1_ = 8, n_2_ = 5, *p* = 0.3407) nor between the 24 and 54 old mice (Mann–Whitney *U* = 13, n_1_ = 5, n_2_ = 10, *p* = 0.1645). Additionally, this test also uncovered a significant increase in motor activity of 24 wks old versus 54 wks old R1098Q mice (Mann–Whitney *U* = 7, n_1_ = 5, n_2_ = 10, *p* = 0.0280). (Figure 1C).

### 3.3. Footprint Test

**Figure 2:**
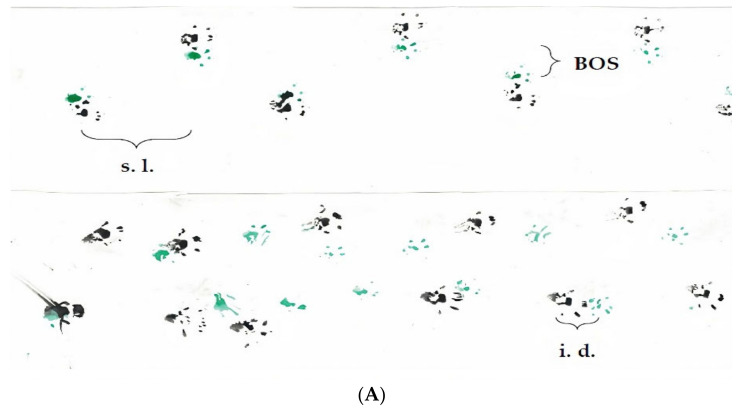

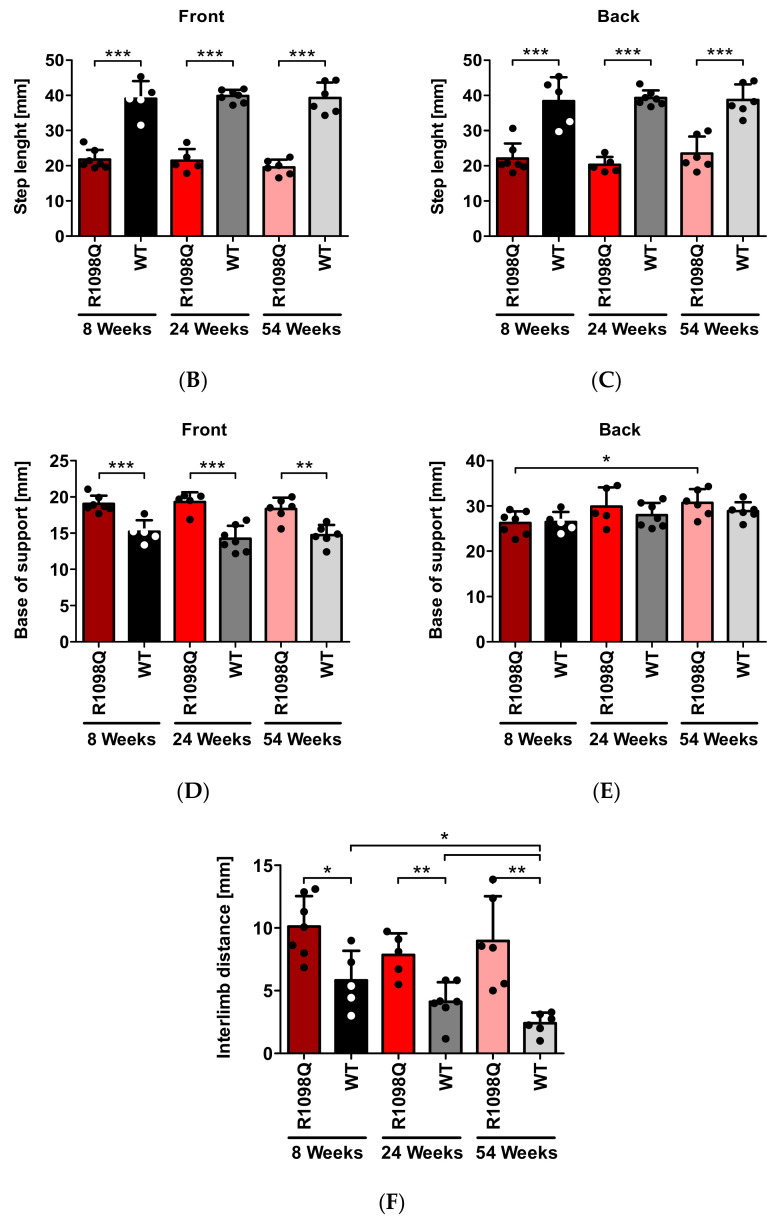
Footprint test. (**A**) Representative footprints of 54-week-old WT and R1098Q littermates (s.l.—step length, i.d.—interlimb distance, and BOS—base of support). Summary data showing a significant decrease in step length (front and back paws), (**B**,**C**) base of support (**D**,**E**), and an increase in the (**F**) interlimb distance between the R1098Q mice and WT controls. The test was performed on the 8-week-old group (WT, n = 5; R1098Q, n = 5), the 24-week-old) group (WT, n = 7; R1098Q n = 7), and the 54-week-old group (WT, n = 6; R1098Q, n = 6). The dots represent the mean measured distances. The bars represent the mean distance achieved by each group. Error bars represent standard deviations (SD). The statistics were calculated using the *t*-test. Statistically significant differences between R1098Q and WT mice, WT mice alone or R1098Q mice alone at different ages indicating the progression of the phenotype are marked with asterisks. (* *p* < 0.05, ** *p* < 0.01, and *** *p* < 0.001).

To assess the gait abnormalities of R1098Q mice, we analyzed the footprint patterns while the mice freely walked along a narrow runway. The footprint patterns were quantified by three measurements: step length, the base of support, and interlimb distance (Figure 2A and Supplementary Figure S2). At all ages the steps of R1098Q mice were unevenly spaced and had their length (both front paws and hind paws) almost twice shorter than WT mice (Figure 2B,C). When analyzed statistically, there was a significant main effect of the genotype (*p* = 0.0001) reflecting differences between the step lengths of R1098Q mice versus the WT littermates. Assessment of the base of support for the front paws showed significantly higher width values, indicative of more splayed and broader steps. This pattern was sustained in all age groups of R1098Q mice as compared with the WT littermates (Figure 2D,E). The same analysis of hind paws showed no significant difference between genotypes, but it revealed a significant extension of width values between 8-week-old and 54-week-old R1098Q mice (*p* = 0.0148). Assessment of interlimb distance provided information on footprint overlap which is a measure of the uniformity of step alterations. Low values typically produced by WT mice (Figure 2F) indicated increasing precision of footsteps with the age of mice. In sharp contrast, almost twice as high values (*p* < 0.05) produced by all age groups of R1098Q mice indicated swayed movements and unsteady, staggering gait.

### 3.4. Wire Hang Test

**Figure 3:**
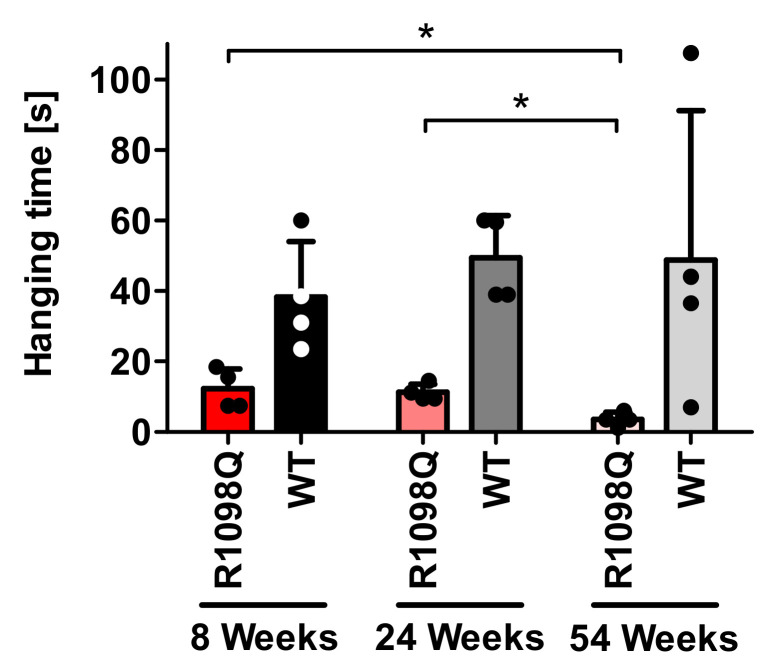
Hanging wire test The graph depicts the difference in hanging time between the R1098Q and WT mice. The 8-week-old group (WT, n = 4; R1098Q, n = 4)), the 24-week-old group (WT, n = 4; R1098Q, n = 4), and the 54-week-old group (WT, n = 4; R1098Q, n = 4). There were 2 females and 2 males in each group. The dots represent the mean hanging time on the wire. Error bars represent standard deviations (SD). The statistics were calculated using the Mann–Whitney test. Statistically significant differences between R1098Q mice at different ages indicating the progression of the phenotype are marked with asterisks. (* *p* < 0.05).

Wire hang test performed to measure muscle strength and endurance revealed that 8, 24, and 54-week-old R1098Q mice had approximately 3, 4.5, and 14.5 times shorter hanging latencies in comparison with WT littermates (*p* < 0.001), respectively (Figure 3). Data also demonstrated the progression of muscle weakness. There was a significant decline in hanging latencies between 24 and 54-wk-old R1098Q mice (Mann–Whitney *U* = 0, n_1_ = 4, n_2_ = 4, *p* = 0.0294), as well as between 8-wk-old and 54-wk-old R1098Q mice (Mann–Whitney *U* = 0, n_1_ = 4, n_2_ = 4, *p* = 0.0294).

### 3.5. Novel Object Recognition

**Figure 4:**
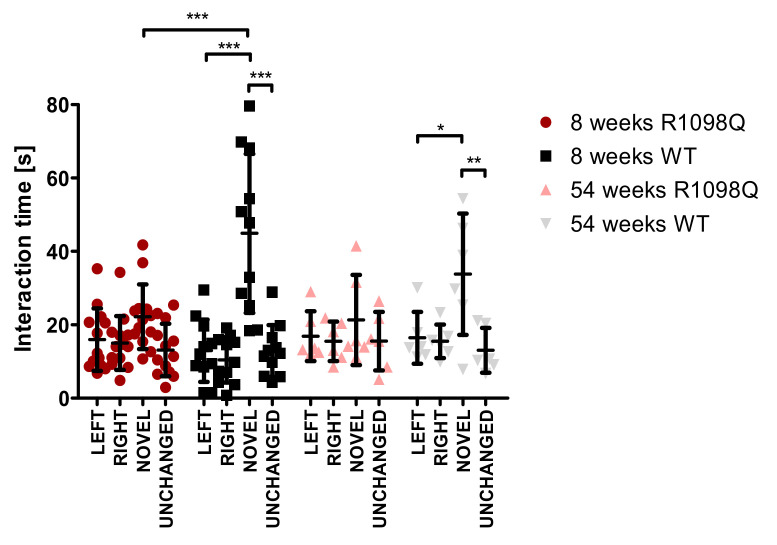
Novel object recognition test. During the first-day training session, two similar objects were used, marked “left” and “right”. On the second day session, one object was replaced by a new one and marked “novel”, while the other object remained the same and was marked “unchanged”. The test was performed on the 8-week-old group (WT, n = 11; R1098Q, n = 13) and the 54-week-old group (WT, n = 6; R1098Q, n = 6). Each point on the graph represents the interaction time of a single mouse with given objects. Error bars represent standard deviations (SD) with the mean interaction time for a given age group marked. The statistics were calculated using two-way ANOVA. Statistically significant differences between R1098Q and WT mice indicating the effect of the mutation on memory are marked with an asterisk (* *p* < 0.05, ** *p* < 0.01, and *** *p* < 0.001).

The novel object recognition test was performed to assess the long-term memory of R1098Q mice [18]. During the training phase, the distribution of exploration time between two objects (left and right) was aproximately 50% irrespective of the age or genotype of mice. (Figure 4). When a novel object was introduced after 24 h, both age groups of WT mice showed a significant increase in exploration time of novel object in comparison to the unchanged object (Figure 4). In contrast, the R1098Q mice, irrespective of their age, did not show significantly increased attention to the introduction of the novel object in comparison to the unchanged object (Figure 4). There was a significantly shorter exploration time of novel object by a group of 8-wk-old R1098Q mice in comparison to WT littermates. This difference could not be extended on 54-wk-old mice mainly because of the shorter exploration times of novel objects presented by old WT mice.

### 3.6. Assessment of Seizure Episodes

**Figure 5:**
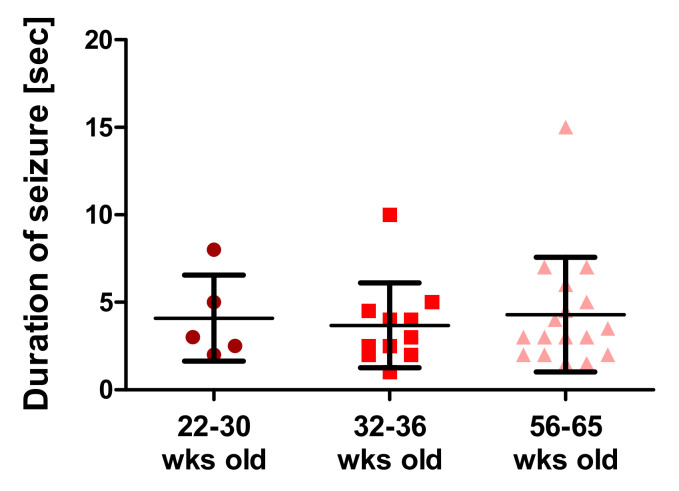
Duration of handling-induced seizures (seconds) in groups of 22–30 wks old (n = 5), 32–36 wks old (n = 11), and 56–65 wks old (n = 17) R1098Q mice. Error bars represent standard deviations (SD) with the mean seizure time for a given age group marked.

The R1098Q mice often presented seizure episodes when handled or after falling off the rotarod. Figure 5 shows the mean length of handling-induced seizures in three age groups of mice. Their average frequency was 75%, and their average duration was around 4 s, irrespective of the age of the mice. Seizure episodes appeared both in males and females at different ages. In the post-recording analysis of movies documenting seizures induced by falling-off rotarod (Supplementary Videos S1–S10 and Supplementary Table S1), we identified clonic and tonic movements while lying on the side, wild jumping, and barrel rolls over the head-tail axis. On the basis of the modified Racine’s scale [17], their pattern was scored as 5–6 on the 7-degree scale.

## 4. Discussion

In this study, we further extended our initial observations regarding the phenotype of R1098Q mice to draw parallels to reported clinical presentations of pathogenic missense *SPTAN1* and *SPTBN* variants in human patients. It is worth noting that although *SPTAN1* variants affecting intrahelical stabilizing interactions within spectrin repeats share a similar proposed pathogenic mechanism, they present a complex spectrum of spastic and ataxia phenotypes. Van de Vondel et al. described a patient bearing the pArg1624Cys variant who presented early-onset cerebellar ataxia, dystonic head movements, and distal muscular atrophy without intellectual disability while other patient carrying pArg1098Gln variant displayed cerebellar ataxia with severe intellectual disability and seizures [1]. Even the same heterozygous *SPTAN1* pAsp1616Asn mutation in three members of the same family, reported by Terrone et al. have a wide spectrum of epilepsy manifestations ranging from benign non-recurrent seizures during mild acute gastroenteritis to early onset generalized epilepsy [19]. Therefore, at present, it seems impossible to a priori predict the severity of phenotype caused by a *SPTAN1* mutation affecting the stability of spectrin repeats. Even within a well-genetically defined R1098Q mouse model, the severity of motor incoordination and lengths of rotarod-induced seizures may differ between littermates (Figure 1, Table S1). This observation can also be extended to other spectrin mutations. For example in the case of three patients carrying the same Arg1003Trp mutation of *SPTBN1* (βII spectrin), only one suffers from seizures and only one has an abnormal brain MRI, while all of them share developmental delay features [9]. In the above example, genetic background, sex, and age differences were proposed to contribute to various disease presentations. However, in the case of inbred R1098Q mice, the variable penetrance of phenotype might also suggest other mechanisms resulting in either uneven expression of *SPTAN1* alleles and/or random pairing of mutated/healthy αII chains with β-spectrins. Another intriguing possibility assumes local instability of mutant αII protein affecting its mechanical properties and interactions with binding partners. Indeed, using thermal circular dichroism we had previously shown that R1098Q-mutant αII peptides had a higher thermal propensity to unfold over a wide range of temperatures [20]. We speculate that physical stressors acting on neurons during synaptic remodeling may cause the premature unfolding of mutant spectrin heterodimers, contributing to neuropathology.

In this study, we also confirmed progressive motor incoordination of R1098Q mice using forced (rotarod) and unforced (grid walking, footprint) walking tests. Progressive ataxia developing in mice older than 3 wks was also reported in βIII spectrin knockout mice (*SPTBN2*) [10]. In contrast to β III knockout mice, however, the grid walking and wire hang tests revealed marked hyperactivity and muscle weakness of R1098Q mice, respectively (Figure 1C and Figure 3). Additionally, animal care operators reported voluntary biting behavior and aggressive behavior of R1098Q mice (data not shown). This compares well to a recent case report by Luongo-Zink et al., who analyzed a 9-year-old patient carrying de novo *SPTAN1* Ser889Cys heterozygous variant [21]. This patient was diagnosed with attention deficit hyperactivity disorder (ADHD) and an autism spectrum disorder. It had self-regulation difficulty that interfered with daily life, including low frustration tolerance, aggression, and impulsivity (kicking, slapping, biting, and pushing). Of interest, ADHD patients carrying autosomal dominant βII variants were recently reported and a similar hyperactivity phenotype in an open field test was also observed in heterozygous βII deficient mice [9]. These data underline common features of a certain heterozygous missense mutation of *SPTAN1* and *SPTBN1* that are well reflected by murine models.

Muscle weakness of R1098Q mice inferred from the poor performance in the wire hang test is reminiscent of the symptoms presented by patients carrying heterozygous *SPTAN1* nonsense mutations reported by Beiyer et al. [3]. Similar to human patients for whom the phenotype is a slowly progressive, juvenile onset, the R1098Q mice show a slow decline of hanging latencies reaching the statistical significance between the groups of adult and old and between young and old mice (Figure 3). As to mechanism behind the peripheral neuropathy caused by dominant negative *SPTAN1* variants, haploinsufficiency caused by nonsense-mediated decay of mRNA encoding mutant alleles has been previously proposed [3]. In the case of R1098Q mice, the allelic imbalance has not been assessed experimentally, nor a possibility of missense-mediated decay reported to affect the splicing efficiency was tested [22]. We only observed that the total expression level of αII in the brains of heterozygous mice did not significantly differ from the wildtype littermates [15]. This result has to be treated with caution because of possible species-specific differences in the degree of transcriptional compensation of the murine *Spna2* (*SPTAN1)* gene [14].

Spatial learning and memory loss are reportedly associated with recurrent seizure episodes in mice [23,24]. Since the R1098Q mice exhibited spontaneous and stress-induced seizures (Figure 5, and Supplementary Table S1), we assessed the spontaneous visual–spatial memory of young and old R1098Q mice using the novel object recognition test. We could find a highly significant difference between the performance of 8-wk-old R1098Q mice in comparison to their WT littermates. This result is consistent with neuronal loss in the neocortex and CA1-CA2 regions of the hippocampus [15]. It also compares well to the reported poor immediate auditory-verbal and visual–spatial memory of the aforementioned 9-year-old SPTAN1 Ser889Cys patient [21].

Taking together, the R1098Q mice present complex phenotype reflecting neuropathology of the central and peripheral nervous systems. For the first time, we document stress-induced seizure episodes and spatial memory impairments in R1098Q mice that compare well to clinical presentations of missense *SPTAN1* variants in humans. Despite genetic homogeneity of R1098Q mice, we still observe an important variability in motor and memory performance, as well as in the severity and duration of seizure episodes between littermates. This observation is consistent with available clinical data regarding variable presentations of the same human missense *SPTAN1* mutation among family members. It could also be extended to other variants of *SPTBN* genes.

## 5. Conclusions

In conclusion, a broad αII spectrin distribution in central and peripheral neurons is a plausible cause of the wide phenotypic spectrum of pathogenic *SPTAN1* variants, reflected in many aspects by the R1098Q mice. In the present study, dominant phenotypic features characterizing the R1098Q mice, namely their unsteady gait and ataxic movements, were extended to reveal hyperactivity, spatial memory impairments, and stress-induced seizure episodes. Our results suggest that all these phenotypic features can be quantitatively assessed when R1098Q mice are to be used as preclinical models for developing genetic and pharmacologic strategies for the treatment of human spectrinopathies.

## Data Availability

The data presented in this study are available upon request from the corresponding author.

## Supplementary Materials

The following supporting information can be downloaded at: https://www.mdpi.com/article/10.3390/brainsci13020261/s1, Figure S1: The rotarod test; Figure S2: Representative images of footprints on the runway tunnel; Table S1: Post recording analysis of rotarod-induced seizures in 6 wks old R1098Q mice; Videos S1–S10: video recordings of rotarod-induced seizures.
