# Supplementary material for: Progressive Ataxia, Memory Impairments, and Seizure Episodes in Spna2 R1098Q Mouse Variant Affecting Alpha II Spectrin’s Scaffold Stability
Source: Brain Sci. 2023 Feb 3;13(2):261. doi: 10.3390/brainsci13020261 (PMC9953789; doi:10.3390/brainsci13020261)
Supplement: Supplementary file 1 [file brainsci-13-00261-s001.zip › brainsci-2170818-supplementary.pdf]

Progressive ataxia, memory impairments, and seizure episodes in *Spna2 R1098Q* mouse variant affecting alpha II spectrin's scaffold stability.

Michał Zalas[1], Joanna Skrzymowska[1], Apolonia Miążek[2], and Arkadiusz Miazek[1,3]

Supplementary Materials: The following supporting information can be downloaded at: www.mdpi.com/xxx/s1, Figure S1: title; Table S1: title; Video S1: title.

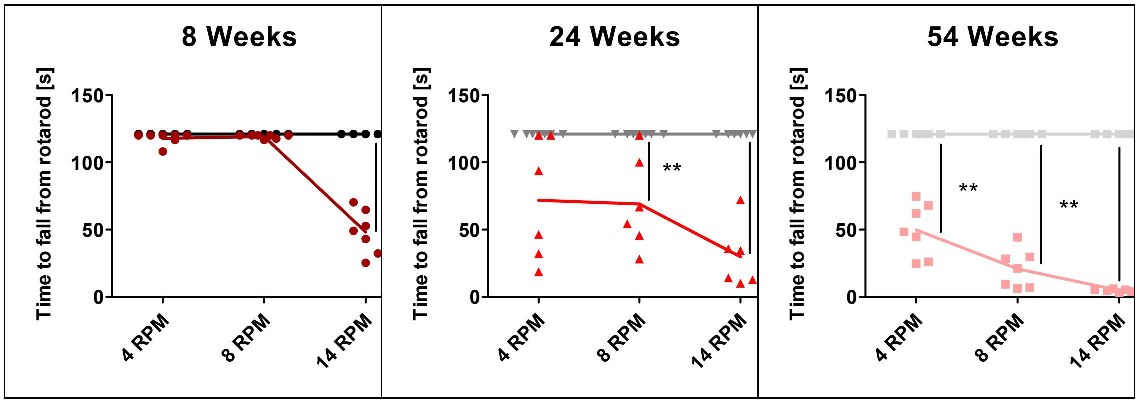

Figure S1. The rotarod test. Differences in times to fall off the constant speed rotarod between age-matched groups of R1098Q and WT controls are shown. Each dot represents the mean latency time the on rotarod achieved by the individual mouse. The 8-, 24, and 54-week-old groups of R1098Q mice are marked in red (●, ●, ●), and WT control littermate groups of mice are marked in black (●, ●, ●) The statistics were calculated using the Mann-Whitney test. Statistically significant differences between R1098Q and WT mice are marked with asterisks. (* p < 0.05, ** p < 0.01, *** p < 0.001)

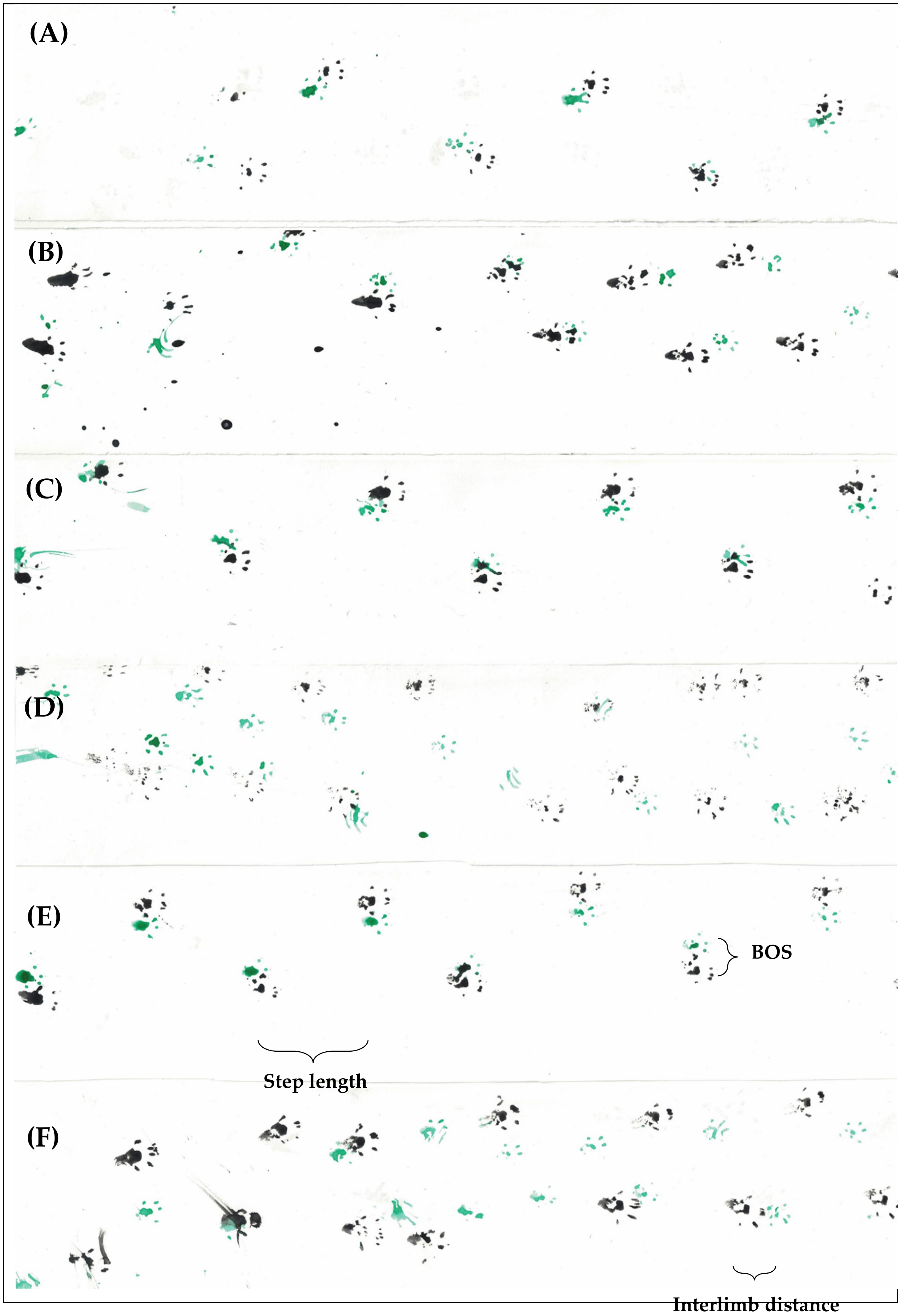

Figure S2. Representative images of footprints on the runway tunnel. The figure shows examples of images recorded for male WT and R1098Q mice from three age groups: **(A)** - 8 week-old WT; **(B)** - 8week-old R1098Q; **(C)** - 24 week-old WT;

(D) - 24 week-old R1098Q; **(E)** - 54 week-old WT; **(F)** - 54 week-old R1098Q. The graph also depicts parameters analysed.

Supplementary video recordings of rotarod-induced seizures:

VID_20201008_135309.mp4 VID_20201008_135608.mp4

VID_20201008_140641_L.mp4 VID_20201008_140641_P.mp4

VID_20201008_141324.mp4

VID_20201008_141728-samice KS i WT kręcą się.mp4

VID_20201008_173000-samce WT+KS spazmy po upadku.mp4

VID_20201008_174931-samica i samiec KS (915 i 921) L.mp4

VID_20201008_174931-samica i samiec KS (915 i 921) P.mp4

VID_20201008_180832-samica KS 917 długi napad.mp4

Table S1`Post recording analysis of rotarod-induced seizures in 6 wks old R1098Q mice

Seizure duration [seconds]	Gender (Female/Male)	Tonic movements	Clonic movements	Barrel rolls/wild jumping
3,5	F	-	+	+
5	F	+	+	+
4	F	+	-	-
13	F	-	+	+
8	F	+	+	+
4	F	+	+	+
6	M	+	+	+
7	F	+	+	+
35	M	+	+	+
65	F	+	+	+
